# Investigation of frequent somatic mutations of MTND5 gene in gastric cancer cell lines and tissues

**DOI:** 10.1080/23802359.2018.1501287

**Published:** 2018-08-29

**Authors:** Lian Li, Rui Xing, Jiantao Cui, Wenmei Li, Youyong Lu

**Affiliations:** a Department of Microbiology, Basic Medical College of Inner Mongolia Medical University, Hohhot, Inner Mongolia, China;; b Laboratory of Molecular Oncology, Key Laboratory of Carcinogenesis and Translational Research (Ministry of Education), Peking University Cancer Hospital & Institute, Beijing, China

**Keywords:** Gastric cancer, mitochondrial ND5 gene, nuclear mitochondrial pseudogenes, single nucleotide variation, single nucleotide polymorphism

## Abstract

The present study investigated the single nucleotide variants (SNVs) in mitochondrial DNA (mtDNA) of 13 paired gastric cancer tissue samples and seven gastric cancer cell lines using direct sequencing analysis of the *MTND5* region. Results showed that nuclear mitochondrial pseudogenes (NUMTs) and mitochondrial copy number affected the detection of the SNV frequency in gastric cancer tissue and cell line samples using high-throughput sequencing technique. The heteroplasmic point mutation C12474T and G12835A happened in AGS and BGC823 cell lines, respectively. A total of seven SNVs were found in three paired gastric cancer tissue samples, including five heteroplasmic point mutations (A12406G, C12705T, T12882C, G12501A, and A12584G) and two homoplasmic point mutations (G12561A and C13590T). Gastric cancer tissue sample 16 exhibited the highest SNVs frequency with four SNVs (np 12406, np 12705, np 12882, and np 12501), whereas no SNVs or SNPs were detected in the tissue sample 4. SNP 12705 turned out to be an SNV in gastric cancer tissue sample 16. SNV 12338 detected by exome sequencing approach appeared to be an SNP in this study.

## Introduction

Mitochondrion is one of the most important organelle in cells that plays important roles in numerous biological activities (Zamzami and Kroemer 2001). For example, it has been confirmed that mitochondrion takes charge of supplying cellular energy through generating adenosine triphosphate (ATP) in cells. Additionally, reactive oxygen species (ROS), one of the most important signalling molecules, are produced and utilized by mitochondria and other organelles in eukaryotes (Sena and Chandel 2012; Kim et al. 2017). More importantly, mitochondria exert an essential role in cellular differentiation and cell death (Galluzzi et al. 2012; Márquez-Jurado et al. 2018).

The mitochondrial genome investigation has suggested that mitochondrion consists of a 16,569 bp circular double-stranded DNA sequence that could encode two rRNAs, 13 respiratory enzyme complex proteins, and 22 tRNAs (Anderson et al. 1981). Reactive oxygen species generated in cells during cell stress could induce damage to cell structures through cleaving lipid, DNA, RNA, and protein (Holzerová and Prokisch 2015). Compared to nuclear DNA (nDNA) in cells, mitochondrial DNA (mtDNA) lacks protective histones and exhibits deficiency on DNA repairing mechanisms (Croteau and Bohr 1997). Therefore, ROS in cells could result in more damage on mtDNA than nDNA, which could cause the mutations of mtDNA (Gu et al. 2016). The accumulation of the mtDNA mutations in cells can decrease its electron transport capacity, which can further decrease the ATP production and increase the level of the ROS. Such mutations could lead to disorders in cell metabolism and thus cause various human diseases (Wallace 2012; Dhillon and Fenech 2014; Lightowlers et al. 2015; Kim et al. 2016). For example, diseases caused by mitochondrial dysfunctions include several types of cancer, Kearns–Sayre syndrome, MELAS syndrome, Parkinson’s disease, Leigh syndrome, myelodysplastic syndrome, diabetes mellitus, and Leber’s hereditary optic neuropathy (Chatterjee et al. 2006; Connolly et al. 2010; Gupta et al. 2013; Montiel-Sosa et al. 2013; Eckenweiler et al. 2015; Coxhead et al. 2016; Kytövuori et al. 2016; Xu et al. 2017).

Gastric cancer is one of the most common cancers all over the world, and it has been confirmed to be the second most common cancer that causes a high mortality rate (Thun et al. 2010; Deans et al. 2011). Gastric cancer incidence has become an increasing trend to Asian population, and it has shown a high mortality rate in China nowadays (Chen et al. 2013). Our preliminary study has revealed that a high mutation rate took place in MTND5 gene in gastric cancer paired clinical tissues using exome sequencing technique, indicating that the *MTND5* mutation could be used as an indicator to estimate the incidence of gastric cancer. Therefore, the present study aimed to validate our hypothesis through analysing mitochondrial single nucleotide variants (SNVs) in gastric cancer tissue and cell lines using polymerase chain reaction (PCR) and Sanger sequencing approaches. The findings from this study could provide practical reference on tumour molecular marker for gastric cancer.

## Materials and methods

### DNA extraction of gastric cancer cell lines and tissue

Gastric cancer cell lines used in this study included BGC823, MGC803, SGC7901, MKN45, AGS, N87, and GES1. The cell lines BGC823, MGC803, and SGC7901 were provided from the Tissue Bank of Shanghai (Shanghai, China). The cell line GES1 was received from the Peking University Cancer Hospital and Institute (Beijing, China). The cell lines MKN45, N87, and AGS were purchased from the American Type Culture Collection (Manassas, VA). These cell lines were cultured in the DMEM medium supplemented with 10% foetal bovine serum in an incubator with 37 °C and 5% CO_2_ condition. Additionally, a total of 13 human gastric cancer specimens and their corresponding adjacent normal tissue samples were provided from the Peking University Cancer Hospital and Institute (Beijing, China). These cancer and adjacent normal tissue samples were collected from the patients during the surgery at the Peking University Cancer Hospital and Institute (Beijing, China). Both gastric cancer cell lines and tissues were extracted using QIAamp DNA Mini Kit to collect genomic DNA according to the manufacture’s instruction (QIAGEN, Hilden, Germany). The genomic DNA (total DNA) in these cell lines and tissues consisted of mtDNA and nDNA. These genomic DNA samples after extraction were immediately stored at –80 °C prior to further analysis.

### Polymerase chain reaction and DNA sequencing

Four primer pairs were designed to amplify the entire length of the mitochondrial ND5 (MTND5) gene using Primer 5.0 Software (Primer Premier, Palo Alto, CA) regarding the NCBI GenBank revised Cambridge Reference Sequence (rCRS) (Anderson et al. 1981; Andrews et al. 1999) and our previous *MTND5* mutations exome sequencing data (Table S1). The detailed information is listed in Table 1. These primers were synthesized from AuGCT Biotech Company (Beijing, China). A total 100 ng DNA amount from each sample was used as a template for the PCR analysis in a 50-µL reaction volume. The PCR condition was carried out as follows: initial denaturation at 94 °C for 5 min, 30 cycles at 94 °C for 30 seconds, annealing at 60 °C for 30 seconds, an extension for *MTND5*-1, 2, 3 and 4 at 72 °C for 30 seconds and 90 seconds for *MTND5* (1–3) and *MTND5* (3–4), and a final extension at 72 °C for 10 min. The PCR products were then analysed using agarose gel electrophoresis and purified through QIAquick PCR Purification Kit according to the manufacture instruction (QIAGEN, Hilden, Germany). The DNA fragments were analysed using direct sanger sequencing under Applied Biosystem 3730XL automatic DNA sequencer (Thermo Fisher Scientific, Waltham, MA) from AuGCT Biotech Company (Beijing, China).

**Table 1.** Primer sequences detecting MTND5 gene.

Primer codes	Primer sequences (5′–3′)	bp
ND5-1		404 bp
ND5-1F	ACTTTTAAAGGATAACAGCTATCC	
ND5-1R	GTCTGAGTTTATATATCACAGTGAG	
ND5-2		310 bp
ND5-2F	ACCTATTCCAACTGTTCATCGG	
ND5-2R	CCTTCTATGGCTGAGGGGAGTC	
ND5-3		471 bp
ND5-3F	TCATAATAGTTACAATCGGCAT	
ND5-3R	TAATGAGAAATCCTGCGAATA	
ND5-4		439 bp
ND5-4F	TATTCGCAGGATTTCTCATTAC	
ND5-4R	ATATTGTAATTGAGATTGCTCG	
ND5 (1–3)		1476 bp
ND5-1F	ACTTTTAAAGGATAACAGCTATCC	
ND5-3R	TAATGAGAAATCCTGCGAATA	
ND5 (3–4)		999 bp
ND5-3F	TCATAATAGTTACAATCGGCAT	
ND5-4R	ATATTGTAATTGAGATTGCTCG	

## Results

### Nuclear mitochondrial pseudogenes

**Figure 1.**
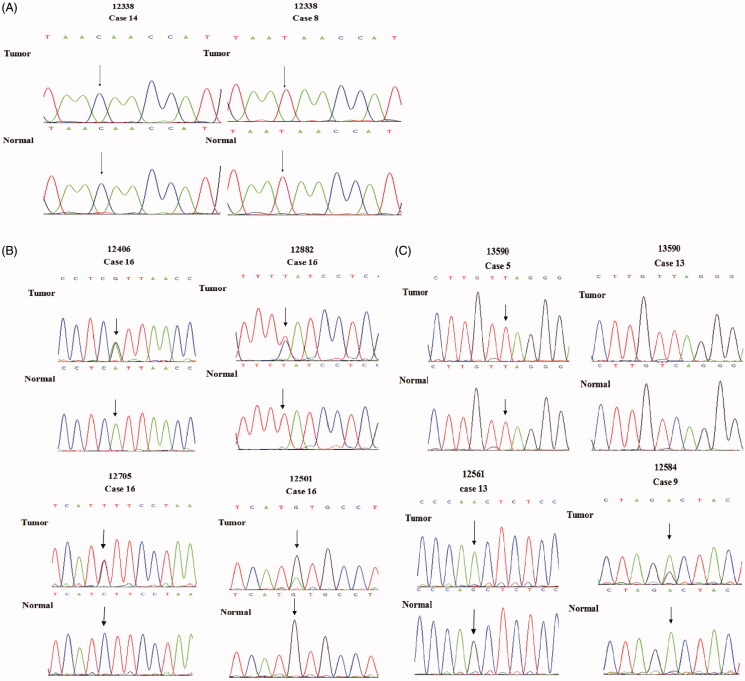
In the sequencing map, SNP and SNV in gastric cancer tissue and adjacent normal tissue. (A) The nucleotide at np 12338 is SNP in present study. In gastric cancer tissue and adjacent normal tissue of No. 14 or No. 8, the nucleotide at np 12338 was the same. There were different nucleotides at np 12338 in No. 14 and No. 8 normal tissue samples. (B) In the sequencing map, double peaks were detected at np 12406, 12882, 12705, 12501. They were SNVs. In No. 16 gastric cancer tissue sample, there are two kinds of nucleotides A and G at np 12406, and there are two kinds of nucleotides C and T at np 12882; T and C were detected at np 12705 in No. 16 gastric cancer tissue; G and A are detected at np 12501 in No. 16 gastric cancer tissue samples; G is detected at np 12501 in normal tissue. (C) It is an SNV at np 13590 in No. 13 paired tissue samples (sequencing map of complementary DNA strand), all of A at np 13590 in normal tissue mutate to A in gastric cancer tissue; in No. 5 paired tissue samples (sequencing map of complementary DNA strand), only A is detected in both of gastric cancer tissue and normal tissue. it is a wildtype at np 13590; G is detected at np 12561 in No. 13 adjacent tissue sample, and whole G mutate to A in gastric cancer tissue sample. G and A are detected at np 12584 in No. 9 gastric cancer tissue samples; A is detected at np 12584 in normal tissue. Part of A at np 12584 mutate to G in gastric cancer tissue sample.

Table S1 shows the preliminary exome sequencing results from 10 paired gastric cancer tissue samples. It was found that a total of 20 SNVs appeared in these five paired samples. Meanwhile, the sequencing results revealed that the SNV sites in the mtDNA were more than that in the nDNA. In the present study, we amplified the entire MTND5 gene from the total DNA in the gastric cancer cell lines and 13 gastric cancer tissue samples. After the PCR amplification, the PCR products were then sequenced. These sequences were searched against the GenBank database. It was found that the *MTND5* from the tissue sample 4 exhibited a 100% and 93% identity with the rCRS and Chr 5 (NC_000005.10, 134926459–134924648) according to the GenBank database, respectively (Figure 1).

It should be worth noting that different nucleotides between the *MTND5* and Chr 5 (134926459–134924648) were interspersed throughout the MTND5 gene. However, no large nucleotide segments were found to be different. Therefore, we further designed the specific primers in the low sequence similarity region between the *MTND5* and Chr 5 to amplify an error-free *MTDN5* gene through the PCR technique. The entire MTND5 gene was screened through merging these two *MTND5* (1–3) and *MTND5* (3–4) segments. In this process, only the MTND5 gene, rather than the Chr 5 (134926459–134924648) gene, was able to be amplified through the primer. In a case that the primers fail to pair the MTND5 gene during the PCR process, the PCR products could still be distinguished and eliminated since they exhibit different gene length compared to the MTND5 gene.

A previous study has suggested that nuclear mitochondrial pseudogenes (NUMTs) could result in the heteroplasmic mutations (Parr et al. 2006). Our amplification method through these primer pairs could ensure the reliable *MTND5* PCR products. It should be noted that the total DNA was used for the exome sequencing analysis. Therefore, false positive results could not be avoided in the tissue samples. This was because since the tissue samples contained large amounts of the mtDNA and nDNA and they could yield high SNVs frequencies due to the similarity of the *MTND5* and Chr 5 sequences. Additionally, the *MTND5* also showed high similarity with the Chr 7, Chr 10, Chr 2, and Chr 4. However, it has been reported that the mtDNA abundance in the tissue samples was much higher than the nDNA, which could cause a higher SNVs frequency in the mtDNA than nDNA (Todorov and Todorov 2009). Therefore, the improvement on the sequencing depth could significantly enhance the reliability of the sequencing results.

### Mitochondrial copy number effect on SNVs frequency

In the present study, we divided the MTND5 gene into four segments, including the *MTND5*-1, 2, 3, and 4. Afterwards, each fragment was amplified using the PCR technique. A BLAST search was applied to the *MTND5*-2 primers and found that the primers exhibited a 100% identity with the *MTND5* sequence. The *MTND5-2* belongs to a segment of the *MTND5* (MT genome 12743–13052), and it showed high identity with the Chr 5 (134926053–134925744). Meanwhile, their amplified products exhibited the same length. The primer ND5-2F has only one nucleotide difference with the Chr 5 (134926053–134926032), and thus annealing temperature could play an important role in avoiding amplification of the Chr 5 during the PCR process. In the present study, we checked the double peak presence in the sequencing analysis. It was found that some different nucleotide sites were specifically identified by the BLAST search between the *MTND5-2* and Chr 5 (134926053–134925744). This could help distinguish these genes on the sequencing analysis.

It has been accepted that same amount of the mtDNA and nDNA could produce same amount of the *MTND5-2* and NUMTs after the PCR. At the same nucleotide position, the different nucleotides from the *MTND5-2* and Chr 5 (134926053–134925744) could produce two peaks on the sequencing analysis. When the amount of the mtDNA in the sample was much greater than the nDNA, the PCR products through the mtDNA template could reach the platform stage with 40 cycles. The NUMTs using the nDNA template could be amplified through 40 cycles during the PCR process. In this study, we used the BGC823 cells as a template, and conducted 40 cycles during the PCR process using the ND5-2F and ND5-2R primers, followed by the PCR products sequencing analysis (Figure S2). It was found that the sequencing result exhibited a 100% similarity to the mtDNA and only one peak was observed in the sequencing analysis. This indicated that the mtDNA accounted for much higher percentage in the total DNA of the BGC823 cells than the nDNA, which resulted in more mtDNA SNVs when the total DNA was used as the template under the exome sequencing approach.

### Single nucleotide polymorphisms (SNPs) in gastric cancer paired tissue samples

Single nucleotide polymorphisms are ubiquitous in gastric cancer tissue and normal tissue (McLean and El-Omar 2014). Therefore, we applied the PCR to amplify the MTND5 gene from the samples, and then sequenced the PCR products using Sanger sequencing to detect the low frequency SNPs (Table S2 and Table 2). It should be noted that the exome sequencing indicated that the np 12338 was an SNV. In the present study, this np 12338 was also detected. The nucleotide at the same position of the MTND5 gene in the gastric cancer tissue sample 14 and the adjacent normal tissue was cytosine. This indicated that no mutation took place at this nucleotide in the gastric cancer sample 14. It should be worth noting that the nucleotide at the np 12338 of rCRS was thymine, the same nucleotide in all other gastric cancer paired tissue samples (Figure 1(A)). This indicated that the np 12338 was an SNP in these tissue samples.

**Table 2.** The result of exome sequencing and sanger sequencing of GC cell lines and paired tissue samples.

		Detected times in exome sequencing/tissue no.	Nucleotide of exome		Nucleotide of tissue			
np of mtDNA	rCRS		Wild	Mutant	Normal	Tumour	Gastric cancer cell line	Amino acid change
12338	T	2/No. 14	C	T	C	C		Met-Thr
12340	A	1	A	G				Thr-Ala
12361	A	2	G	A				Ala-Thr
12406	G	1/No. 16	A	G	A	G/A		Ile-Val
		2	G	A			GES1 A	Val-Ile
12572	G	1	G	A				Ser-Asn
12882	C	2	C	T	T	T/C	GES1 T	Phe-Phe
		1/No. 16	T	C				
13353	A	1	A	G				Leu-Leu
13368	G	1	G	A				Gly-Gly
13708	G	1	G	A				Ala-Thr
13759	G	1	A	G			GES1 A	Thr-Ala
		2	G	A				Ala-Thr
13928	G	1	G	C			GES1 C	Ser-Thr
14120	C	1	T	C				Leu-Pro
12705	C	No. 13, 9, 8, 6, 1, 2			T	T	BGC823, N87	Ile-Ile
							MGC803	
		No. 16			C	C/T	SGC7901, MKN45 T	
12372	G	No. 6, 9			A	A	AGS A	Leu-Leu
12584	A	No. 9			A	A/G		Asp-Gly
12720	A	No. 1			G	G		
13563	A				G	G		
13933	A				G	G		
13263	A	No. 2			G	G		
13681	A	No. 3			G	G		Thr-Ala
13269	A	No. 3, 7			G	G		
12358	A	No. 9			G	G		Thr-Ala
13093	A	No. 5			G	G		
13590	G	No. 5			A	A		
		No. 13			G	A		Leu-Leu
12588	C	No. 12			T	T		
13145	G	No. 2, 12			A	A		
13395	A	No. 12			G	G		
12561	G	No. 13			G	A		Gln-Gln
12696	T	No. 14			C	C		
12501	G	No. 16			G	G/A		Met-Met
12950	A				G	G		
12474	C						AGS C/T	Ile-Ile
12771	G						N87 A	Glu-Glu
12835	G						BGC823 G/A	Ala-Thr
13105	A						N87, BGC823 G	Ile-Val
13830	T						N87 C	Leu-Leu
13914	C						BGC823 A	Leu-Leu

^a^ Some same sense mutations are not shown in the table of amino-acid change; G and A represent guanine and adenine, respectively. G/A means two nucleotides (G and A) in tissue samples.

Additionally, thymine was found at the np 12705 in five gastric cancer cell lines except for the GES1 and AGS (Table 2), whereas the nucleotide at the same position in the rCRS was cytosine. It was found that thymine was detected at the np 12705 in some gastric cancer tissues and normal tissues (sample 1, 2, 6, 8, 9 and 13), the nucleotide at same position in the other paired tissue samples appeared to be cytosine. This indicated that the np 12705 was also an SNP. Similarly, numerous SNPs were identified in the tissue samples using the same analysis, including A12720G in the tissue sample 1, T13263C in the tissue sample 2, A12950G, T13681C and A13269G in the tissue sample 3, A13093G and G13590A in the tissue sample 5, G12372A in the tissue sample 6 and 9, A13269G in the tissue sample 7, A12358G in the tissue sample 9, and T12696C in the tissue sample 14. It should be noted that thymine was the nucleotide at the np 12705 in the cell line BGC823, N87, MGC803, SGC7901, and MKN45, whereas the nucleotide at the np 12372 in the AGS cell line was adenine. Their nucleotide was not as the same as that in the rCRS. However, the 12705 and 12372 could not be determined to be an SNP in these cell lines due to the lack of the control cells.

### Single nucleotide variants in gastric cancer tissue and cell lines

A total of seven SNVs were found in three samples out of 13 gastric cancer paired tissue samples, indicating an approximate 23% mutation rate in the present study. Additionally, the mtDNA mutation frequency appeared to be different in these three gastric cancer tissue samples. For example, an SNV was detected in every 453 bp in the tissue sample 16, whereas the tissue sample 14 exhibited an SNV frequency at every 906 bp. Only one SNV was found in the tissue sample 9. It should be noted that six SNVs found in these tissue samples were found to be the SNPs regarding the dbSNP database. However, the nucleotide at the position np 12584 appeared to be a novel SNV in these tissue samples.

It was observed that both thymine and cytosine were present at the np 12882 position in the gastric cancer tissue sample 16 with the similar quantity (Figure 1(B)). However, the nucleotide at the np 12882 position in the adjacent normal tissue sample 16 was found only to be thymine, whereas cytosine was the only nucleotide at the np 12882 of the rCRS. This indicated that an SNV happed at the np 12882. The T12882C was a heteroplasmic point mutation. Such a mutation could further lead to a synonymous mutation of amino acids.

Meanwhile, the exome sequencing data revealed that the nucleotide at the np 12340 was mutated from adenine to guanine in the gastric cancer tissue samples 210. The nucleotide at the np 12340 position was detected 96 times with adenine 22 times and guanine 74 times, indicating that two different nucleotides at the np 12340 position were found in the gastric cancer tissue samples. The A12340G was also a heteroplasmic point mutation (Table 1).

The nucleotide at the np 12406 in two tissue samples (GC208 and GC209) was mutated from guanine to adenine, whereas the GC205 tissue sample had the nucleotide at this position was guanine instead of adenine. In the gastric cancer 208 normal tissue sample, the nucleotide at the np 12406 position was detected 76 times with adenine 12 times and guanine 64 times. The sanger sequencing analysis indicated that two peaks (guanine and adenine) were observed at the np 12406 in the gastric cancer tissue sample 16 (Figure 1(B)). However, only adenine peak was found at the np 12406 position in the normal tissue sample 16. These indicated that adenine in the gastric cancer tissue sample was mutated from guanine at the np 12406 in some mtDNA, which could further result in the mutation of amino acid formation from isoleucine to valine. In the gastric cancer cell lines, adenine and thymine were found at the np 12406 and 12882 position in the gastric cancer cell line GES1, respectively.

In the gastric cancer sample 16, double peaks were found at the np 12705 position representing thymine and cytosine (Figure 1(B)). However, cytosine was the only nucleotide found at this position in the normal tissue sample 16. This indicated that the mtDNA might be partially mutated from cytosine to thymine at this position in the gastric cancer tissue 16, since those double peaks at the np 12705 were similar as the SNVs at the np 12406 and 12882. This also suggested that the nucleotide at the np 12705 turned out to be an SNV in the gastric cancer paired tissue sample 16, although it was an SNP in other tissues and previously published reports (Mosquera-Miguel et al. 2009; Ren et al. 2015). Similarly, the nucleotide at the np 13708 has been reported to be an SNP (van der Walt et al. 2003; Mosquera-Miguel et al. 2009). In the exome sequencing analysis, guanine and adenine were found at this position for 11 and 13 times in the normal tissue samples. However, the nucleotide at the np 13708 was found to be adenine in the gastric cancer tissue sample. This indicated that the nucleotide at the np 13708 appeared to be an SNV in the exome sequencing analysis.

It should be worth noting that some SNVs were not identified using the exosome sequencing analysis. However, they were detected via the PCR and Sanger sequencing analyses in the present study. For example, the nucleotide at the np 12584 position in the gastric cancer tissue sample 9 consisted of adenine and guanine, whereas adenine appeared to be the nucleotide at this position in the adjacent normal tissue sample. These indicated that some mtDNA in the gastric cancer tissue sample 9 was mutated from adenine to guanine, which further resulted in an alteration on amino acid synthesis from aspartic acid to glycine. Similarly, the gastric cancer tissue sample 16 exhibited guanine and adenine at the np 12501 position, whereas the nucleotide at the same position in the adjacent normal tissue sample was guanine. This caused a synonymous mutation in the gastric cancer tissue sample 16.

A homoplasmic point mutation was found at the np 13590 position in comparison of the gastric cancer tissue sample 13 with its adjacent normal tissue sample. Particularly, the nucleotide at this position in the normal tissue sample was guanine, whereas it was mutated to adenine in the gastric cancer tissue sample 13. Such a mutation could further lead to a synonymous mutation on amino acids. It should be noted that adenine was the only nucleotide at the np 13590 position in the gastric cancer tissue sample 5 and its adjacent normal tissue sample (Figure 1(C)), whereas the nucleotide at the same position in the other paired tissue samples was guanine. These indicated that the nucleotide at the np 13590 in the cancer tissue sample 13 was an SNV but it was an SNP in the other tissue samples. Additionally, the nucleotide at the np 12561 also turned to be a homoplasmic point mutation. This was because adenine and guanine were found to be the nucleotide at this position in the gastric cancer tissue sample 13 and its adjacent tissue sample, respectively (Figure 1(C)).

In the gastric cancer cell lines, two heteroplasmic point mutations were also found, including the C12474T in the AGS cell and the G12835A in the BGC823 cell. The mutation of G12835A resulted in the amino acid formation from alanine to threonine (Table 2). In addition, the comparison on the sequence of these gastric cancer cell lines with the rCRS exhibited the differences in seven nucleotides. For example, the nucleotide at the np 13759 and 13928 were adenine and cytosine in the GES1 cell line, whereas the difference in the N87 cell line was observed in adenine at the np 12771, cytosine at the np 13830, and guanine at the np 13105. The nucleotide at the np 13914 and 13105 in the BGC823 cell line appeared to be adenine and guanine, respectively.

## Discussion

We hypothesized that gastric cancer incidence might result from the mtDNA mutation, and such mtDNA mutations might be caused by mitochondrial SNVs in gastric cancer tissue. After analysing paired gastric cancer tissue samples and gastric cancer cell lines using a combined approach of the PCR and Sanger sequencing analyses, nine *MTND5* SNVs and some SNPs were detected. This included seven MTND5 SNVs found in the 13-paired gastric cancer tissue samples and two SNVs in the gastric cancer cell lines. More importantly, the point mutation frequency appeared to be different in paired gastric cancer tissue samples from different patients. For example, the SNV at the position 12406, 12705, 12882, and 12501 was found in the gastric cancer tissue sample 16 compared to its adjacent normal tissue sample using the PCR technique. No SNVs and SNPs were observed in the gastric cancer tissue sample 4. Besides, we also found that the nucleotide at the position 12406, 12705, 12501, 12882, 12561, and 13590 appeared to be SNVs in the gastric cancer tissue and cell line samples of the present study although they have been classified as the SNPs in the dbSNP database. It has been reported that SNPs could also trigger the carcinogenesis (Wang et al. 2007; Grzybowska-Szatkowska and Ślaska 2014; Williams Stephen et al. 2015; Bussard and Siracusa 2017). For instance, SNPs G9055A, A10398G, and T16519C have been reported to increase the breast cancer risk (Bai et al. 2007). It has also been suggested that SNP 10398G played an inherited predisposition factor in developing the breast cancer (Czarnecka et al. 2010). Our results were in an accordance with these studies.

Exome sequencing analysis could determine the nucleotide at the specific position of a mutation. It should be noted that a nucleotide with the high frequency could be considered the mutated nucleotide in the site, whereas the low frequency nucleotide could be ignored when a site contains two nucleotides. The present study could indicate that the same tissue or cell could contain two different types of mtDNA, which was consistent with the previous study (Ye et al. 2014). Furthermore, we proposed that the mtDNA point mutations in the present study might result from a process where a normal mtDNA was gradually mutated by a mutated mtDNA in cells/tissues. When the mtDNA mutations reached to a certain level, the mutated cells might become cancerous (McCann et al. 2015; Stefano and Kream 2016).

## Conclusions

In conclusion, NUMTs and mitochondrial copy number exerted an important role in affecting the detection of SNV frequency in gastric cancer tissue and cell line samples using high-throughput sequencing technique. The *MTND5* point mutation frequency appeared to be different in different gastric cancer tissue and cell line samples. Heteroplasmic point mutation C12474T and G12835A were found in AGS and BGC823 cell lines, respectively. A total of seven SNVs were observed in three paired gastric cancer tissue samples, including five heteroplasmic point mutations (A12406G, C12705T, T12882C, G12501A, and A12584G) and two homoplasmic point mutations (G12561A and C13590T).

## Abbreviations

MT-ND5: NADH dehydrogenase subunit 5 of mitochondrial DNA
SNP: Single nucleotide polymorphism
SNV: Single nucleotide variant
NUMTs: Nuclear mitochondrial pseudogenes
BLAST: Basic local alignment search tool
rCRS: Revised Cambridge reference sequence
PCR: Polymerase chain reaction
np: Nucleotide position
ROS: Reactive oxygen species
